# Benign causes of diffusion restriction foci in the peripheral zone of the prostate: diagnosis and differential diagnosis

**DOI:** 10.1007/s00261-016-0719-6

**Published:** 2016-04-12

**Authors:** Nirjhor M. Bhowmik, Jinxing Yu, Ann S. Fulcher, Mary A. Turner

**Affiliations:** Department of Radiology, Virginia Commonwealth University Health System, Main Hospital, 3rd Floor, 401 North 12th Street, P.O. Box 980615, Richmond, VA 23298 USA

**Keywords:** Prostate, Cancer, Diffusion restriction, Multiparametric-MRI, Prostatitis, Hypertrophic nodule, Displaced central zone, Post-biopsy hemorrhage, Granulomatous prostatitis

## Abstract

Multiparametric-MRI is an important tool in the diagnosis of prostate cancer (PCa), particularly diffusion-weighted imaging for peripheral zone (PZ) cancer in the untreated prostate. However, there are many benign entities that demonstrate diffusion restriction in the PZ mimicking PCa resulting in diagnostic challenges. Fortunately, these benign entities usually have unique MR features that may help to distinguish them from PCa. The purpose of this pictorial review is to discuss benign entities with diffusion restriction in the PZ and to emphasize the key MR features of these entities that may help to differentiate them from PCa.

Prostate cancer (PCa) is the most frequently diagnosed form of non-cutaneous malignant tumor in men and the second leading cause of cancer death in men [1]. It is estimated that one in six men will be diagnosed with prostate cancer in their lifetime [2]. Current clinical screening methods such as prostate-specific antigen (PSA) testing or trans-rectal ultrasound (TRUS)-guided prostate biopsies for PCa lack sensitivity and specificity [3, 4]. In recent years, multiparametric-MRI (mp-MRI) has emerged as an important tool in the diagnosis of PCa and as a means in assisting targeted biopsy, risk stratification, and treatment selection [5]. These advancements in mp-MRI are mainly attributed to the addition of diffusion-weighted imaging (DWI) and dynamic contrast enhancement (DCE) to the routine prostate MR protocol [5]. However, a wide variety of normal and abnormal entities with diffusion restriction in the peripheral zone (PZ) can mimic PCa on mp-MRI and create diagnostic challenges [6, 7].

The adoption of Prostate Imaging Reporting and Data System (PI-RADS) has been instrumental in promoting a greater level of standardization and consistency with regards to the acquisition, interpretation, and reporting of prostate mp-MRI examinations [8]. According to PI-RADS v2, the dominant sequence for detection of PCa in the PZ is DWI-ADC map. This statement has gained a lot of support from experienced prostate MRI readers, although it is important to keep in mind that many mimics may have falsely high PI-RADS scores because of their diffusion restriction.

Since our department performs MRI-guided and US/MRI fusion-guided prostate biopsies, we have gained extensive experience in differentiating benign from malignant foci that demonstrate diffusion restriction in the PZ. Benign entities with diffusion restriction in the PZ include but are not limited to chronic prostatitis, hypertrophic nodule in the PZ, normal displaced central zone, insertion of capsule and fascia at the midline of the PZ, post-biopsy hemorrhage, thickened surgical capsule, enlarged neurovascular bundle, granulomatous prostatitis, ejaculatory ducts, and prominent periprostatic fat. Recognition of the MR findings of these benign entities that demonstrate diffusion restriction in the PZ, along with the key MR features that may help to differentiate them from PCa, is important in avoiding unnecessary interventions and guiding clinical management.

## Prostate cancer in the peripheral zone

**Fig. 1:**
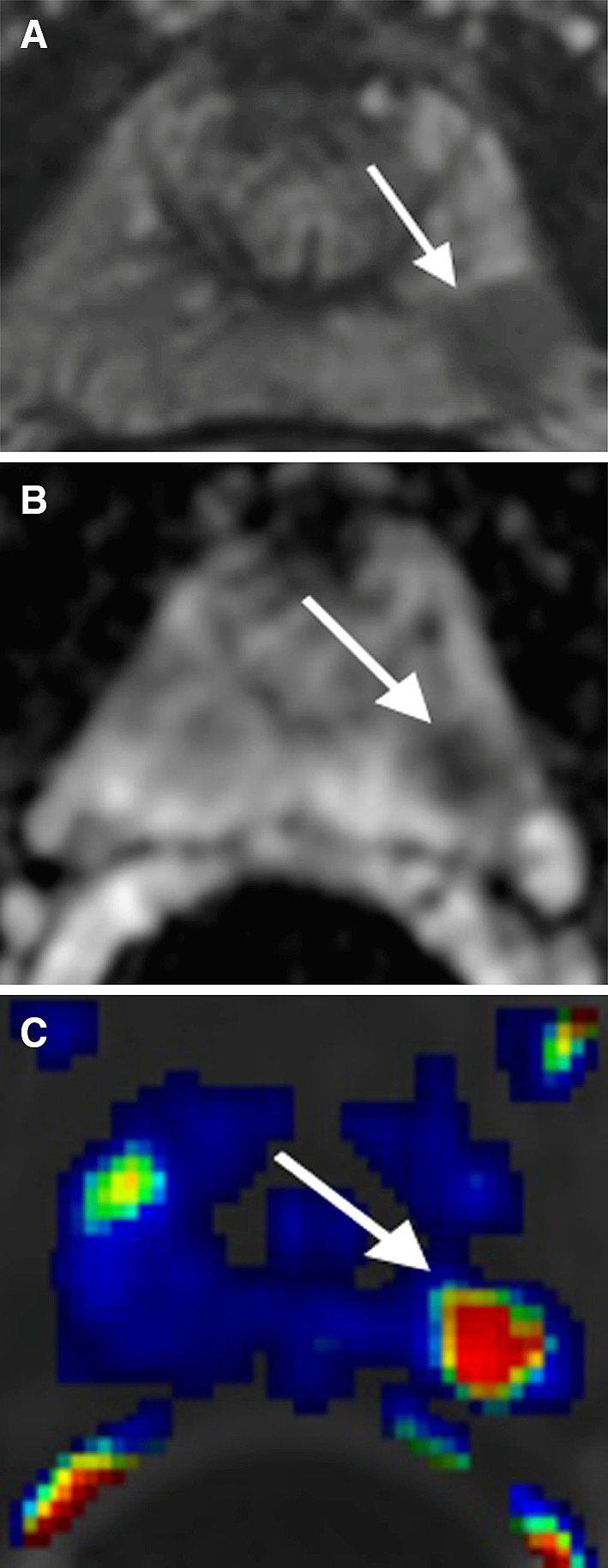
67-year-old male with PCa Gleason score 7 in the left apex PZ confirmed by US/MRI fusion biopsy. **A** Axial T2WI shows a large hypointense T2 signal lesion with mass effect in the left apex PZ (*arrow*). **B** ADC map shows diffusion restriction of the lesion (*arrow*). **C** DCE demonstrates rapid contrast wash-in and wash-out of the lesion (*arrow*)

PCa in the PZ typically manifests as a hypointense signal focus on T2-weighted imaging (T2WI) and apparent diffusion coefficient (ADC) map with early contrast wash-in and wash-out on dynamic contrast-enhanced imaging (DCE) (Fig. 1) [5]. At times, PCa may result in mass effect on the adjacent normal prostate tissue and prostate capsule. The ADC value correlates with the PCa Gleason score such that a lower ADC value is usually associated with higher Gleason scores [8, 9]. Diffusion restriction within a lesion raises suspicion for PCa (Fig. 1B) [10, 11]. Typically, an early and avidly enhancing lesion that exhibits >20% rapid wash-out of contrast during the 4-min time interval after intravenous contrast administration will be red on most available DCE images after software processing (Fig. 1C) [7], raising the suspicion for PCa. If an area in the prostate does not exhibit wash-out, it will be blue in color. A green area in the prostate after software processing indicates wash-out of contrast between 0% and 20%. If an institution does not have DCE as part of its routine protocol, the diagnostic accuracy of PCa may still be maintained, particularly for experienced prostate MRI readers, even though DCE may increase the sensitivity and specificity for the diagnosis of PCa.

## Chronic prostatitis

**Fig. 2:**
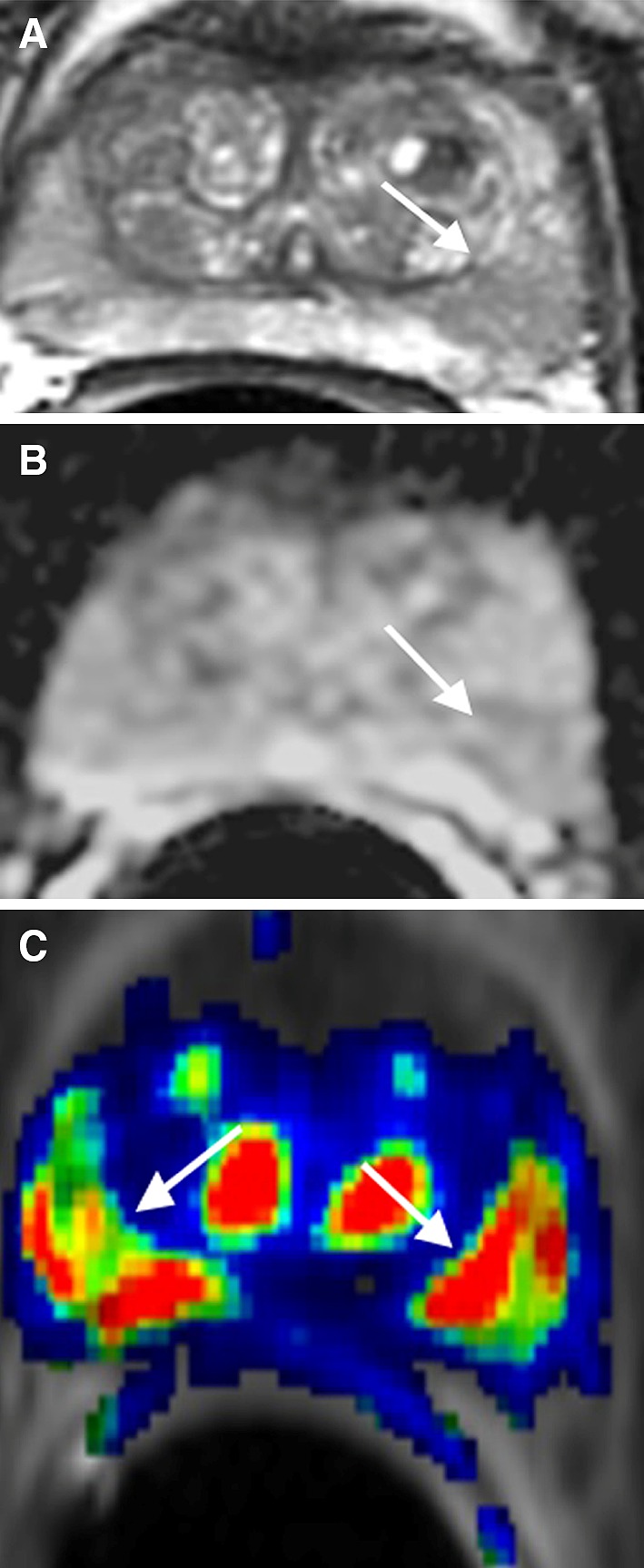
52-year-old male with chronic prostatitis in the left mid PZ confirmed by MRI-guided biopsy. **A** Axial T2WI shows a hypointense T2 signal focus in the left mid PZ (*arrow*) without mass effect. **B** ADC map demonstrates mild diffusion restriction of the focus (*arrow)* in the left mid PZ. **C** DCE shows enhancement of the focus, similar to the right mid PZ enhancement (*arrows*)

Chronic prostatitis is one of the most common benign entities that mimic PCa in the PZ. It may be diffuse or focal in the PZ with hypointense T2 signal, diffusion restriction, and abnormal enhancement, mimicking PCa (Fig. 2) [12, 13]. Despite the overlap of these MR characteristics with PCa, there are clues that help differentiate chronic prostatitis from PCa. On T2WI, the hypointense T2 signal areas in chronic prostatitis are usually geographic, ill-defined, and lack the contour deformity or mass effect on the adjacent normal prostate tissue or capsule that is sometimes seen with PCa [14]. On DCE, these areas may show bilateral symmetric rapid contrast wash-in and wash-out usually in a band-like or wedge-shaped morphology [8]. Most importantly, the degree of diffusion restriction of chronic prostatitis is often less than that seen in PCa, providing crucial evidence that this is not PCa (Fig. 2B) [13, 14].

## Hypertrophic nodule in the peripheral zone

**Fig. 3:**
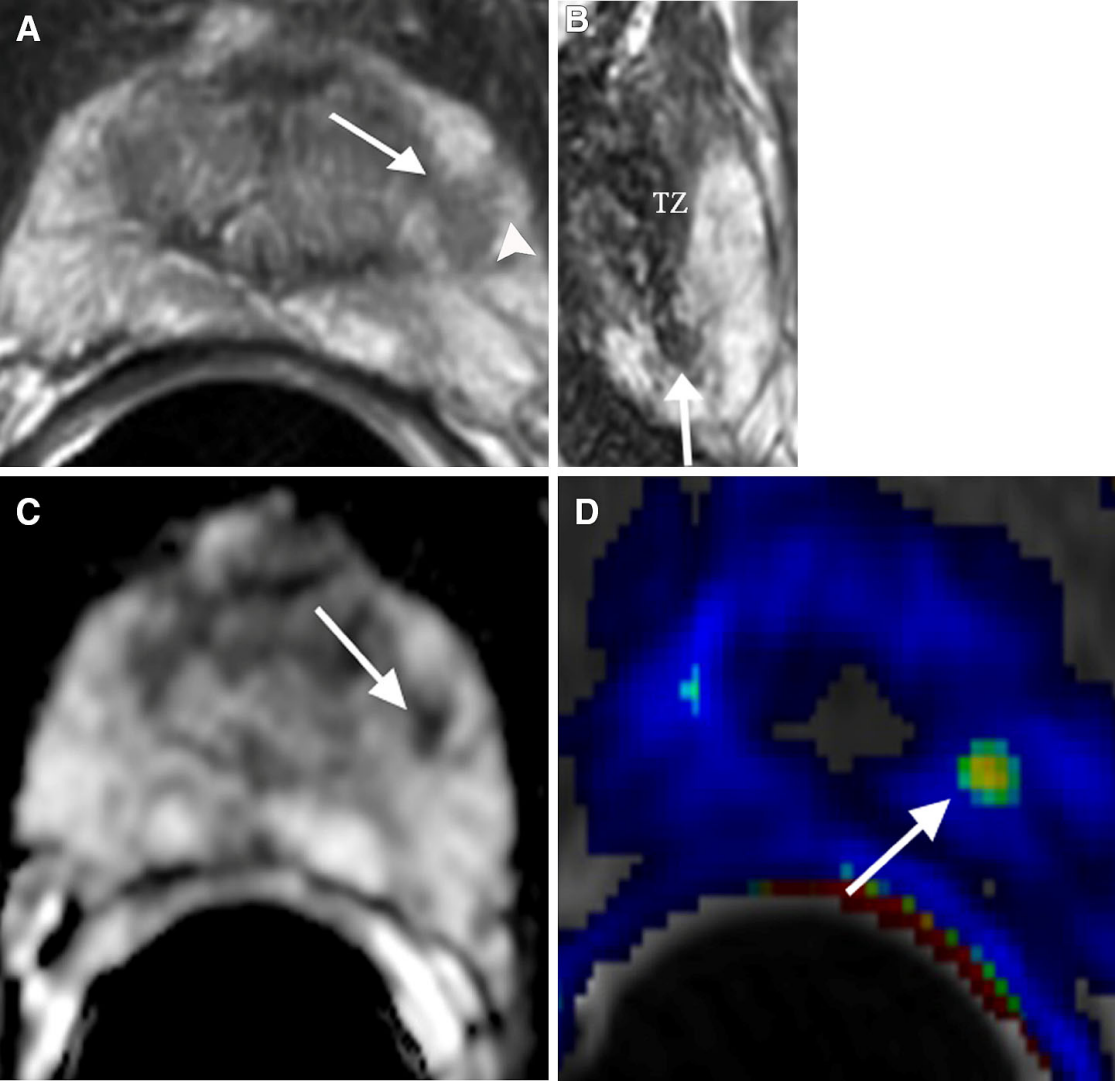
66-year-old male with hypertrophic nodule in the left mid PZ confirmed by US/MRI fusion biopsy. **A** Axial T2WI shows a well-defined hypointense signal focus (*arrow*) in the left mid PZ with a layer of normal prostate tissue (*arrowhead*) between the lesion and capsule. **B** Sagittal T2WI shows that the well-defined focus (*arrow*) is continuous with the adjacent central gland (CG). **C** ADC map shows diffusion restriction of the focus (*arrow*) mimicking PCa. **D** DCE shows rapid enhancement with wash-out of the focus (*arrow*)

Hypertrophic nodules in the PZ have hypointense T2 signal, diffusion restriction, and rapid contrast wash-in and wash-out, mimicking PCa (Fig. 3) [14]. However, these nodules are usually less than 1 cm in size and rounded or oval in shape with discrete, well-defined margins and with thin T2 hypointense encapsulation [8]. Since most of these nodules arise from the transitional zone, they are often continuous with the adjacent transitional zone in one of three planes on T2WI, which provides strong MR imaging evidence of a benign nodule [15]. Hypertrophic nodules usually do not extend to the capsule on T2WI, so that normal tissue between the lesion and the capsule is more often seen as compared to that in PCa (Fig. 3A) [14, 16].

## Normal displaced central zone

**Fig. 4:**
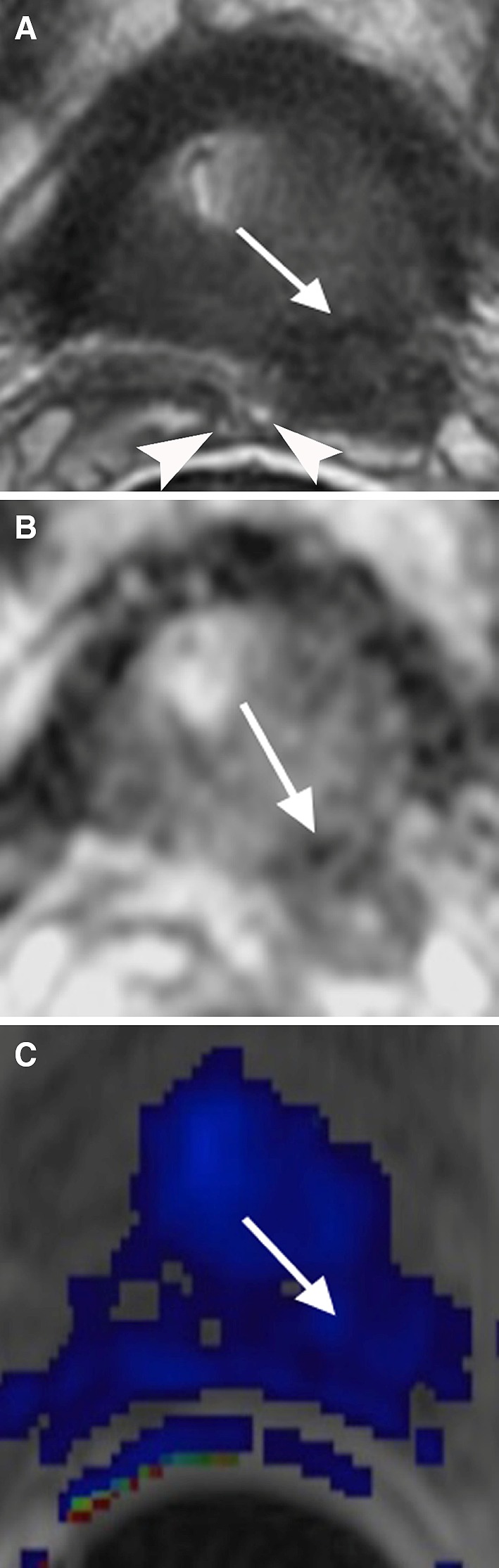
68-year-old male with displaced central zone in the left base PZ confirmed by MRI-guided prostate biopsy. **A** Axial T2WI shows a hypointense signal area in the left base PZ (*arrow*) at the level of ejaculatory ducts (*arrowheads*). **B** ADC map shows diffusion restriction of the area (*arrow*) mimicking PCa. **C** DCE shows no significant contrast enhancement with wash-out of the area (arrow)

Current zonal anatomy of the prostate describes three distinct glandular regions: peripheral zone, transition zone, and central zone. The central zone appears as a symmetric band of tissue between the peripheral and transition zones, extending posterior to the transition zone and urethra, proximal to the verumontanum, and surrounding the ejaculatory ducts [17, 18]. The central zone exhibits homogenously hypointense T2 signal and low ADC value relative to the PZ. When the transition zone is hypertrophic, it can compress and displace the central zone superiorly and laterally to the base, just inferior to the seminal vesicles [14]. If this process results in asymmetry of the right or left central zone, the displaced central zone can be misinterpreted as PCa (Fig. 4). However, on T2WI, the central zone is often visualized at the level of the ejaculatory ducts with sharp margins (Fig. 4A). Further, the central zone demonstrates no or minimal rapid enhancement with wash-out on DCE (Fig. 4C), in contrast to PCa with rapid wash-in and wash-out [19].

## Insertion of capsule and fascia at the midline of the peripheral zone

**Fig. 5:**
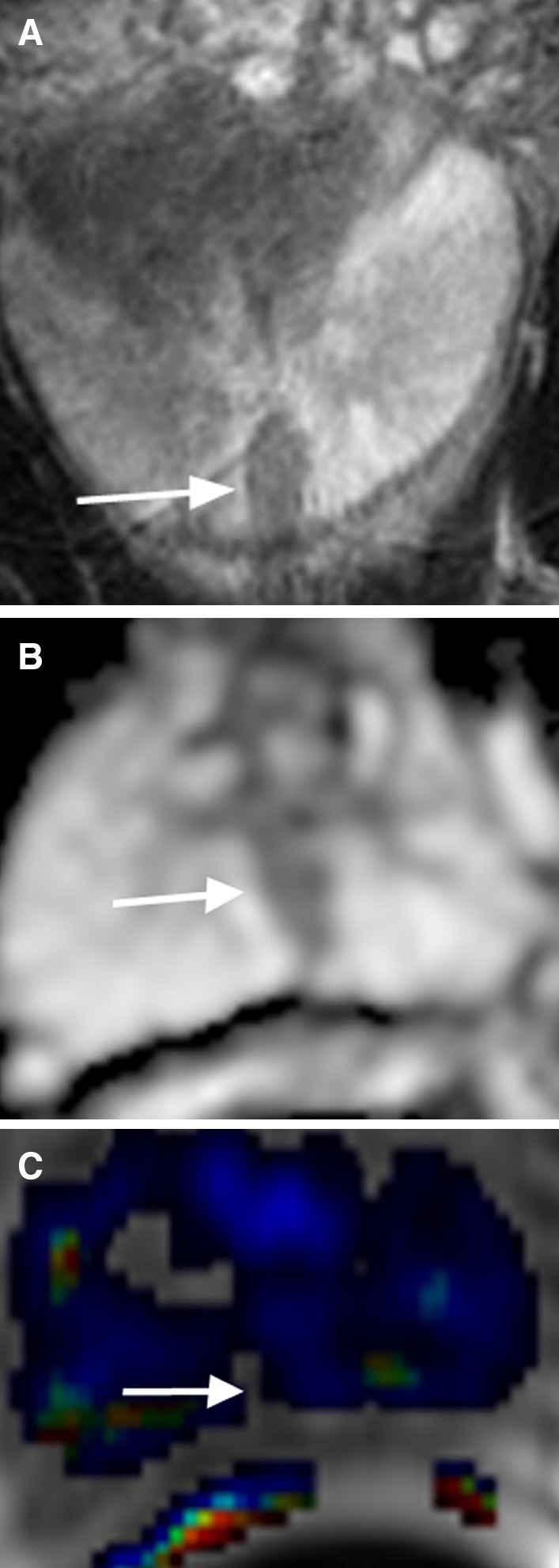
73-year-old male with a “pseudolesion” secondary to insertion of capsule and fascia in the midline of the PZ confirmed by MRI-guided biopsy. **A** Coronal T2WI shows a hypointense signal region (*arrow*) in the midline of the PZ. **B** ADC map shows diffusion restriction of the region (*arrow*) mimicking PCa. **C** DCE demonstrates no enhancement of the region (*arrow*)

A hypointense T2 signal focus at the midline of the peripheral zone at the level of the midgland to apex may occasionally be identified. It is believed that fusion of the prostatic capsule and fascia at this region is responsible for the hypointense T2 signal focus [20]. This midline hypointense T2 signal focus may show diffusion restriction on the ADC map, mimicking PCa (Fig. 5). MRI features including its midline location, concave contour of the prostate at the focus, and lack of dynamic contrast enhancement are the key clues for differentiating this entity from PCa [16].

## Post-biopsy hemorrhage

**Fig. 6:**
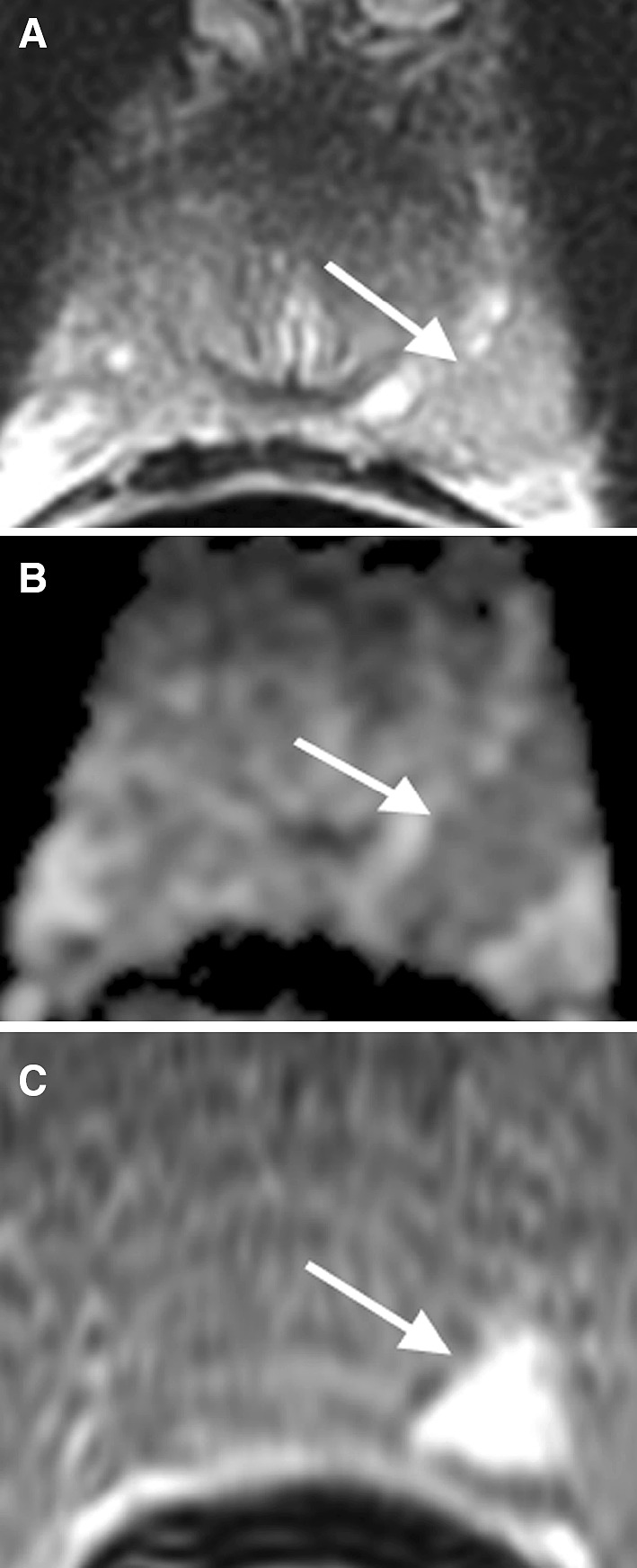
56-year-old male with post-biopsy hemorrhage in the left apex of the PZ. **A** Axial T2 shows a hypointense T2 signal region in the left apex of the PZ (*arrow*) with mass effect mimicking PCa. **B** ADC map shows mild diffusion restriction of the region (*arrow*). **C** Axial T1 shows hyperintense signal in the region (*arrow*) consistent with hemorrhage

 Citrate is found in fairly high concentrations in healthy prostate epithelium and prostatic fluid. In addition to its role as a preservative in semen, citrate also functions as an anticoagulant [5]. This property may contribute to the presence of hemorrhage within the PZ for an extended time after prostate biopsy [21]. Specifically, hemorrhage may cause hypointense T2 signal, diffusion restriction, and abnormal contrast enhancement, mimicking PCa (Fig. 6) [22]. Hemorrhage may also obscure underlining tumor [14, 21], thus further complicating the diagnosis. Key clues for differentiating post-biopsy hemorrhage from PCa include mild diffusion restriction on ADC map and hyperintense signal of the area on T1WI [21–23]. Additionally, due to decreased levels of citrate in PCa, the degree of hemorrhage in discrete PCa is often significantly less than the surrounding benign prostate tissue. This “hemorrhage exclusion sign,” whereby prostate tumors are outlined by extensive surrounding hemorrhage on T1WI, may aid in tumor localization [24]. A post-biopsy delay of at least 6 weeks or longer is recommended before performing a prostate MRI in order to allow enough time for the resorption of blood products [8, 14].

## Thickened surgical capsule

**Fig. 7:**
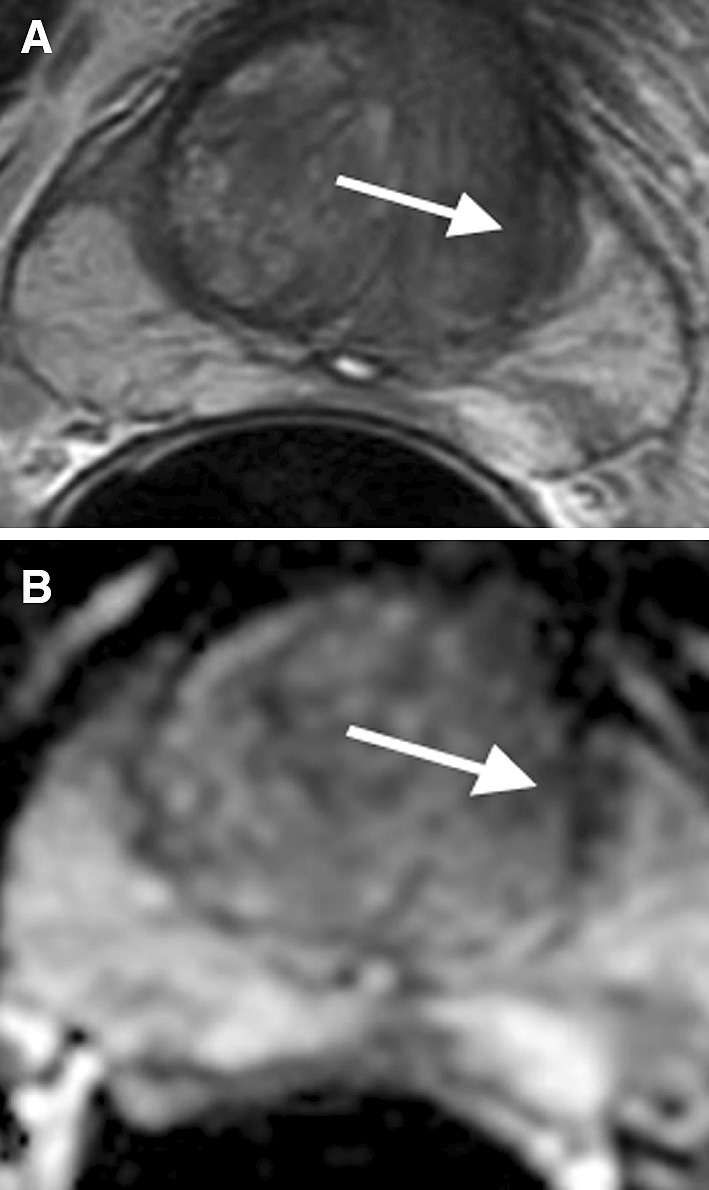
68-year-old male with thickened left surgical capsule mimicking PCa. **A** Axial T2 shows a band-like hypointense T2 signal area between the left transitional and peripheral zones (*arrow*). **B** ADC map shows diffusion restriction of the area (*arrow*)

In addition to the anatomic capsule of the prostate that surrounds the PZ, a “surgical” capsule surrounding the transition zone has also been described, since it provides a landmark for benign prostatic hypertrophy (BPH) surgery. This structure arises from the embryologic periurethral septum and is composed of fibrous and muscular tissue [25]. Outward pressure from the development of BPH within the transition zone induces proliferation and thickening of this fibromuscular layer between the transitional and peripheral zone. On MRI, the surgical capsule appears as an elongated hypointense T2 signal area with low ADC value mimicking PCa (Fig. 7A, B). However, the classic location of the surgical capsule (between the transitional zone and PZ) with a band-like or elongated shape can serve as a clue to differentiate it from PCa [26]. Further, as the capsule is simply fibromuscular tissue, there is no dynamic contrast enhancement with wash-out on DCE images.

## Enlarged neurovascular bundle

**Fig. 8:**
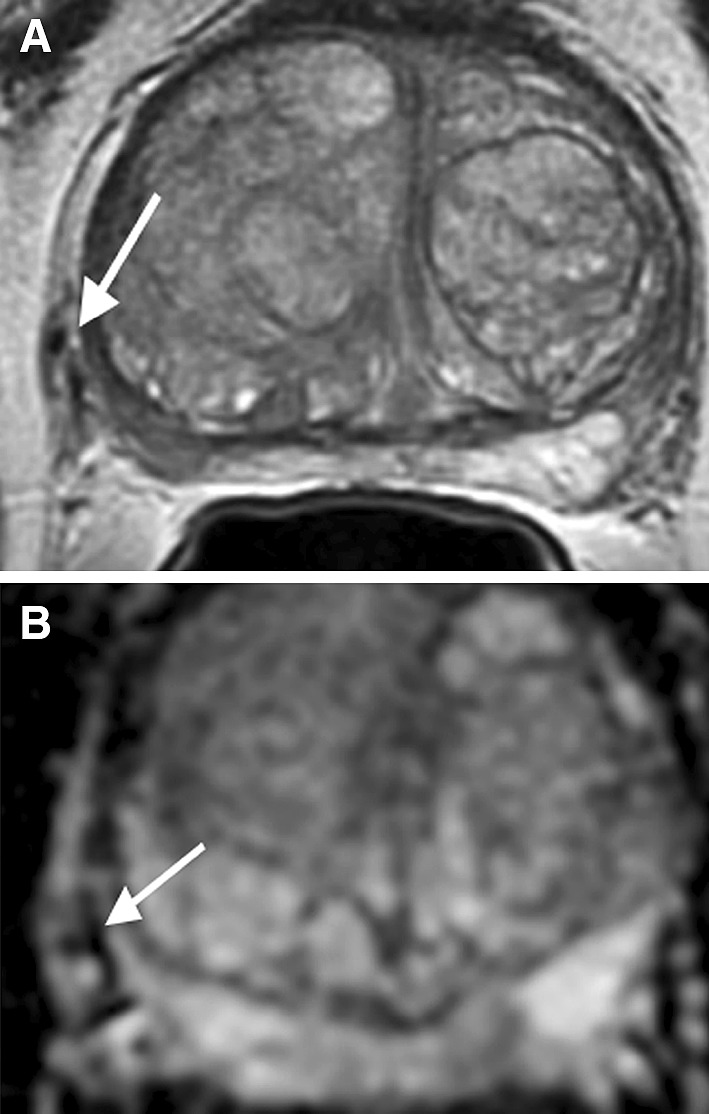
58-year-old male with prominent neurovascular bundle in the right base. **A** Axial T2WI shows hypointense T2 signal foci along the right base in the lateral aspect of the PZ (*arrow*). **B** ADC map shows diffusion restriction of the foci along the right base of the PZ (*arrow*) mimicking PCa

The neurovascular bundle (NVB) includes the nervous plexus, arteries, veins, and additional smaller nerve branches that supply the prostate gland. It has classically been viewed as a discrete structure coursing along the posterolateral margin of the prostate near the prostate capsule at approximately the 5- and 7-o’clock positions [27]. The structure exhibits hypointense signal on T2WI and ADC map. Due to the proximity of the NVB to the PZ, the discrete rounded appearance of the NVB may be mistaken for PCa (Fig. 8). Key clues in differentiating the NVB from PCa include its typical location along the outer edge of the prostate capsule and tubular appearance when tracked across multiple consecutive slices [27–29].

## Granulomatous prostatitis

**Fig. 9:**
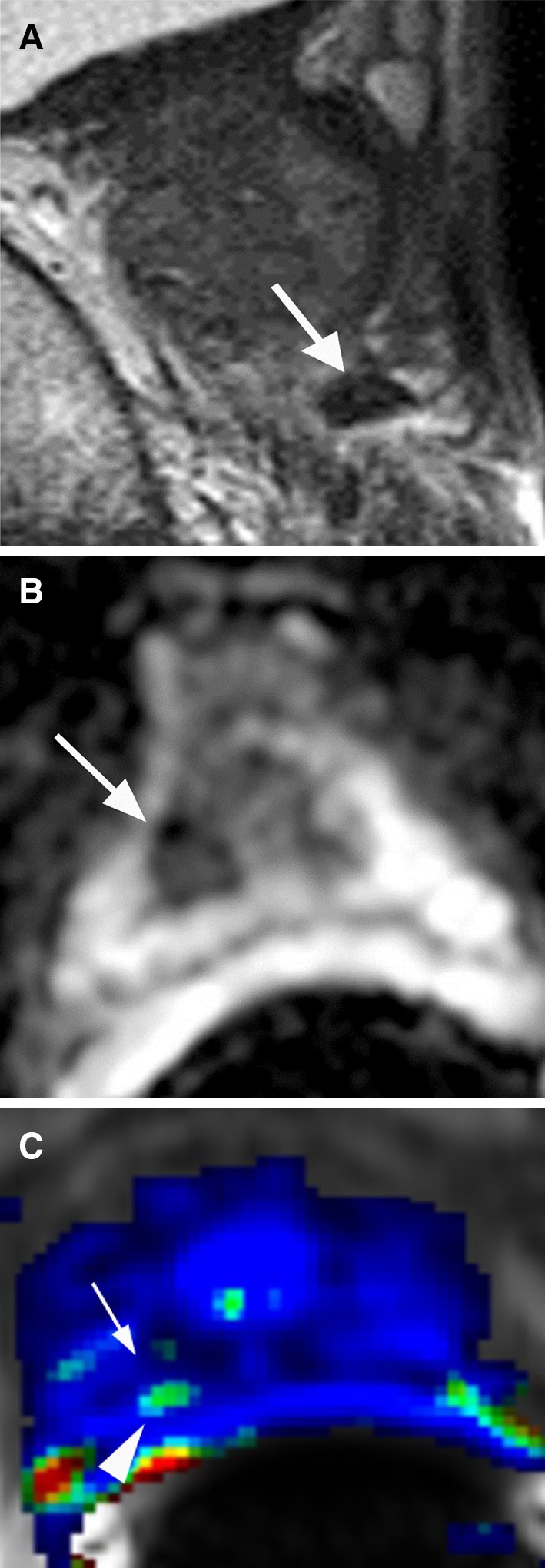
71-year-old male with granulomatous prostatitis in the right apex PZ confirmed by US/MRI fusion biopsy. **A** Sagittal T2WI shows a hypointense lesion in the right apex PZ (*arrow*). **B** ADC map shows diffusion restriction of the lesion (*arrow*). **C** DCE demonstrates mild enhancement with wash-out only in the posterior aspect of the lesion (*arrowhead*) and the majority of the lesion (*arrow*) without enhancement

Granulomatous prostatitis is an uncommon benign inflammatory condition that clinically mimics PCa, since it often presents as a firm nodule on digital rectal exam with elevated PSA [14]. On MR imaging, this entity appears as a discrete mass with significant hypointense signal on T2WI and ADC map mimicking PCa (Fig. 9). Additionally, as this is an inflammatory process, there may be associated infiltration of the periprostatic fat, which can appear similar to extraprostatic tumor extension [30]. Currently, histopathologic analysis is the conclusive method in excluding the presence of underlying tumor. However, correlation with clinical history including a rapid, progressive course of disease and prior bacilli Calmette–Guérin (BCG) immunotherapy is useful when considering granulomatosis prostatitis as a diagnosis [14, 30]. Another clue in differentiation is that granulomatous prostatitis demonstrates large areas without enhancement due to foci of necrosis within the lesion [31]. In one study, caseating necrosis was identified in 76% of cases of infectious granulomatous prostatitis [32].

## Ejaculatory ducts

**Fig. 10:**
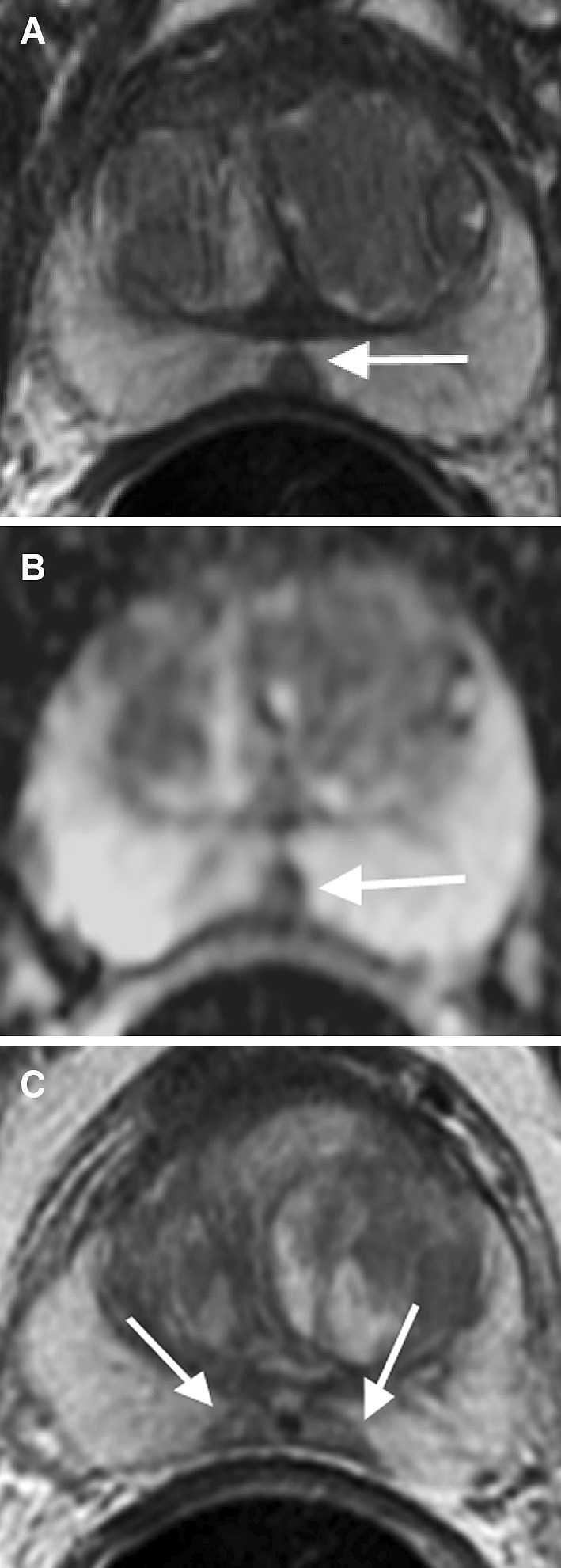
67-year-old male with prominent ejaculatory ducts. **A** Axial T2WI shows a hypointense T2 signal focus at the midline PZ (*arrow*) at the level of the midgland. **B** ADC map shows diffusion restriction of the focus (*arrow*) at the midline of the PZ mimicking PCa. **C** Axial T2WI superior to (**A**) shows the typical bilateral, symmetric ejaculatory ducts (*arrows*) excluding PCa

The ejaculatory ducts are paired tubules that originate near the vas deferens behind the prostate and next to the seminal vesicles. They course through the prostate and empty into the prostate urethra at the verumontanum. The ducts are visualized in the medial aspect of the PZ near the midline at the level of the base to midgland. Since the ducts are encased by intermittent bundles of longitudinal fibers, they can present as hypointense signal on T2WI and ADC map, mimicking PCa (Fig. 10) [5, 33]. Their typical location, bilateral, and elongated appearance, and lack of rapid contrast wash-in and wash-out on DCE help in differentiating them from PCa.

## Prominent periprostatic fat

**Fig. 11:**
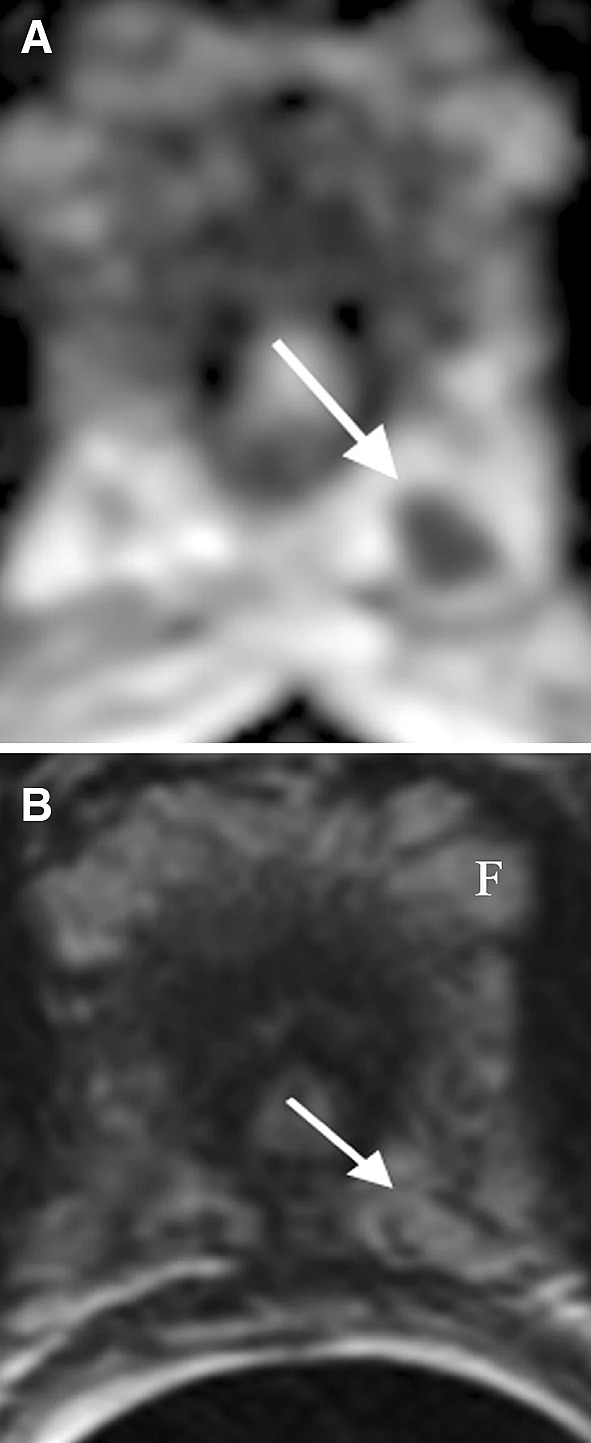
61-year-old male with prominent periprostatic fat. **A** ADC map shows diffusion restriction of an area (*arrow*) near the left apex of the PZ mimicking PCa. **B** Axial T2 shows hyperintense T2 signal in the corresponding focus (*arrow*), identical to that of the adjacent fat (*F*) excluding PCa

One of the shortcomings of diffusion-weighted imaging include susceptibility-induced distortions. Susceptibility effects from chemical shift artifacts caused by periprostatic fat can lead to significant diffusion restriction which mimics PCa (Fig. 11A) [5]. However, unlike PCa, fat will demonstrate hyperintense T1 and T2 signal (Fig. 11B) and will not enhance. Another clue in differentiation is that periprostatic fat is located adjacent to but outside the prostate [5, 27].

## Summary

Although there have been substantial improvements in the accuracy of MRI for the detection of PCa, achieving optimal accuracy can be hindered by many mimics, which create even more difficulty in interpretation for inexperienced prostate MRI readers. In this article, we have demonstrated that a wide variety of benign entities with diffusion restriction in the PZ can mimic PCa. However, these entities also have their own MR features that help to distinguish them from PCa (Table 1). Ultimately, recognition of the unique MR features of these mimics on mp-MRI, along with additional clues that help to differentiate these entities from PCa, is important in establishing a correct diagnosis and guiding clinical management.

**Table 1:** Key MR clues that help in differentiating mimics from prostate cancer

Benign causes of diffusion restriction foci in the PZ	Key clues in differentiation
Chronic prostatitis	Ill-defined margins with no contour deformity Slight diffusion restriction Sometimes symmetric contrast wash-in and wash-out bilaterally
Hypertrophic nodule in the PZ	Round or oval with well-defined margins Thin T2 hypointense encapsulation Often continuous with adjacent transitional zone on T2WI Layer of normal tissue between nodule and prostate capsule
Normal displaced central zone	Commonly symmetric and well defined At the level of ejaculatory ducts No or minimal rapid contrast wash-in and wash-out
Insertion of capsule and fascia at the midline of the PZ	Midline location with concave contour of the prostate at the focus No rapid contrast wash-in or wash-out
Post-biopsy hemorrhage	Mild diffusion restriction Hyperintense T1 signal “Hemorrhage Exclusion Sign”
Thickened surgical capsule	Band-like or crescentic shape At the junction of the PZ and transitional zone No rapid contrast wash-in or wash-out
Enlarged neurovascular bundle	Tubular appearance on at least one plane Located along outer edge of the prostate capsule
Granulomatous prostatitis	Large areas of non-enhancement due to foci of necrosis within the lesion
Ejaculatory ducts	Located at the medial aspect of the PZ near the midline from the base to midgland Bilateral and elongated appearance
Prominent periprostatic fat	Hyperintense T1 and T2 signal Adjacent to but outside the prostate No rapid contrast wash-in or wash-out
